# Metabolic Pathways of *Leishmania* Parasite: Source of Pertinent Drug Targets and Potent Drug Candidates

**DOI:** 10.3390/pharmaceutics14081590

**Published:** 2022-07-30

**Authors:** Surbhi Jain, Utkarsha Sahu, Awanish Kumar, Prashant Khare

**Affiliations:** 1 Department of Microbiology, All India Institute of Medical Sciences, Bhopal 462026, Madhya Pradesh, India; 315surbhijain@gmail.com (S.J.); utkarsha.sahu1@gmail.com (U.S.); 2 Division of Synthetic Biology, Absolute Foods, Plot 68, Sector 44, Gurugram 122003, Haryana, India; 3 Department of Biotechnology, National Institute of Technology, Raipur 492010, Chhattisgarh, India

**Keywords:** *Leishmania*, human pathogen, targeting metabolic pathways, repurposed drugs, new anti-leishmanials

## Abstract

Leishmaniasis is a tropical disease caused by a protozoan parasite *Leishmania* that is transmitted via infected female sandflies. At present, leishmaniasis treatment mainly counts on chemotherapy. The currently available drugs against leishmaniasis are costly, toxic, with multiple side effects, and limitations in the administration route. The rapid emergence of drug resistance has severely reduced the potency of anti-leishmanial drugs. As a result, there is a pressing need for the development of novel anti-leishmanial drugs with high potency, low cost, acceptable toxicity, and good pharmacokinetics features. Due to the availability of preclinical data, drug repurposing is a valuable approach for speeding up the development of effective anti-leishmanial through pointing to new drug targets in less time, having low costs and risk. Metabolic pathways of this parasite play a crucial role in the growth and proliferation of *Leishmania* species during the various stages of their life cycle. Based on available genomics/proteomics information, known pathways-based (sterol biosynthetic pathway, purine salvage pathway, glycolysis, GPI biosynthesis, hypusine, polyamine biosynthesis) *Leishmania*-specific proteins could be targeted with known drugs that were used in other diseases, resulting in finding new promising anti-leishmanial therapeutics. The present review discusses various metabolic pathways of the *Leishmania* parasite and some drug candidates targeting these pathways effectively that could be potent drugs against leishmaniasis in the future.

## 1. Introduction

Leishmaniasis is a tropical disease endemic to Asia, Northern Africa, the Middle East, the Mediterranean, and South and Central America. According to the World Health Organization (WHO), 1.5 to 2 million new cases and 70,000 deaths due to *Leishmania* infection are observed annually worldwide. The disease is caused by the digenetic and intracellular protozoa *Leishmania* spp. (approximately 21 pathogenic species) and is generally transmitted by infected sandflies (*Phlebotomus* or *Lutzomyia* spp.) [1]. The parasite occurs in two forms, namely, amastigote and promastigote. Amastigotes (axenic and intracellular) remain at a low rate of proliferation, a reduced biosynthetic capacity, a lower bio-energetic level, and a significantly changed metabolism. The parasites are taken along with the blood meal from an infected person by a female sand fly [2]. Parasites change from amastigotes to procyclic promastigotes within the sand fly, then grow into virulent (metacyclic) promastigote forms. Mammalian hosts are infected with the promastigote form of the parasite [3]. Leishmaniasis may cause cutaneous, mucocutaneous, or visceral disease. Cutaneous leishmaniasis (CL) is the most prevalent type of leishmaniasis. It causes skin lesions on the body, leaving life-long scars as well as major impairments [2]. Another form of leishmaniasis is mucocutaneous leishmaniasis (MCL), which causes the mucous membranes of the nose, mouth, and throat to be partially or completely destroyed. CL and/or MCL are caused by *L. major* and *L. tropica*, as well as *L. amazonensis*, *L. braziliensis*, and *L. mexicana*. In visceral leishmaniasis (VL) caused by *L. donovani*, *L. infantum*, and *L. chagasi*, the major targets are the visceral organs, including the liver, spleen, and bone marrow. The common symptoms of VL include fever, splenomegaly, hypergammaglobulinemia, and pancytopenia [4].

Despite the fact that leishmaniasis is a terrible condition, the treatment options are restricted and unsatisfactory. Currently, the major treatment options against leishmaniasis mainly consist of chemotherapy, where the drugs are toxic, costly, have limited administration routes, and have multiple side effects. Drugs such as antimony sodium stibogluconate, amphotericin, paromomycin, and oral drug miltefosine [5] are currently administered to counter *Leishmania* infections. These medications, in general, have some drawbacks, such as the need for long-term treatment, parenteral administration, harsh side effects, high cost, and the evolution of resistant parasites [6]. Consequently, there is a persistent need to discover novel, cheap, and highly potent anti-leishmanial drugs with pharmacokinetics features. Different metabolic pathways of the *Leishmania* parasite are involved in its proliferation and growth. Pathways such as sterol biosynthetic, purine salvage, glycosyl phosphatidyl inositol (GPI) biosynthesis, folate biosynthesis, hypusine, and trypanothione pathway play a crucial role in parasite survival and proliferation [7]. Thus, the crucial role of these pathways in parasites makes them vital drug targets to counter *Leishmania* infection.

Repurposing already approved medications to treat different diseases is becoming an effective option. This method offers various advantages, including the ability to use compounds with known pharmacological effects and avoiding the high costs and extended research timelines associated with developing new leishmanicidal drugs [8]. Although the strategy of examining medications with other approved uses to uncover alternative anti-leishmanial treatments are relatively new, there are several viable candidates with promise in vitro and in vivo leishmanicidal capabilities [9]. The process of developing new indications for existing, failing, or abandoned medications or advanced clinical candidates is known as drug repurposing [10]. Several drug repurposing programs have been started in recent years, with a focus on neglected tropical and uncommon diseases [8,11,12]. The initial step in the drug development method is to choose a target in the parasite biochemical pathway that is either completely absent in the host or distinct from the host homolog [13]. Furthermore, the desired parasite target should be involved in critical metabolic pathways or cellular functions, whose obstruction results in parasite death or growth suppression [14]. Moreover, now that genome libraries are available, known pharmacological modes of action can be matched in silico to pathogen-specific gene targets, resulting in possible therapeutic candidates [15,16].

Herein, we have reviewed the major metabolic pathways in *Leishmania* spp. as a target to seek useful drug repurposing approaches for the treatment of leishmaniasis. Comprehensive knowledge of potential drug targets within the metabolic pathways of the *Leishmania* parasite would be beneficial in reducing the mortality and morbidity associated with *Leishmania* sp. infection. In addition, the drug repurposing approach would also solve the problem of time and cost constraints associated with the discovery of new drugs.

## 2. Drug Candidates Targeting Metabolic Pathways of *Leishmania*

### 2.1. Sterol Biosynthetic Pathway

**Figure 1:**
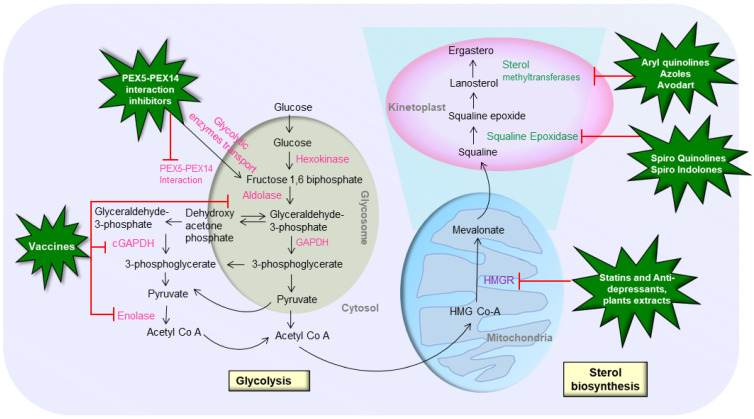
Potential drug targets and repurposed drugs against glycolysis and sterol biosynthesis pathways of *Leishmania* parasite.

In the human pathogenic stages of several trypanosomatid parasites, such as *Leishmania*, sterol production has been reported to be a key metabolic process. Sterols are key components in eukaryotic cells that regulate membrane fluidity and permeability. They play a role in the cell cycle and development as a precursor and regulator [17]. the enzymes of this pathway are being researched extensively because of distinct differences from their mammalian counterparts [18]. The pathway creates ergosterol in *Leishmania*, which is replaced by cholesterol in the host. Conventional anti-leishmanial drugs such as amphotericin B, miltefosine, and azoles work by disrupting the permeability barrier of the cell membrane by inhibiting or impairing the sterol biosynthesis pathway [19,20]. Repurposing drugs that also target the sterol biosynthesis pathway could potentially lead to the discovery of novel anti-leishmanial drugs. The initial stage in sterol biosynthesis is squalene epoxidation, which is catalyzed by squalene epoxidase (SE). Then, as an initial precursor for the biosynthesis of all steroid structures, the squalene epoxide intermediate is transformed into lanosterol [21]. The main steroid lanosterol goes through a series of changes resulting in cholesterol (in mammals) and ergosterol (in trypanosomatids and fungi). To date, different potential compounds have been reported that can target the sterol biosynthetic pathway and can be repurposed to counter leishmaniasis (Figure 1).

Spiro-indolone NITD609 E is a promising antimalarial drug candidate [22], and spiro[cyclohexanone-oxindoles]-like molecule can inhibit *L. infantum* promastigote and axenic amastigote proliferation in a dose-dependent manner [23]. Furthermore, spiro[indole-3,3′-pyrrolizidine]-2-one has recently been discovered to operate as a catalytic inhibitor of *L. donovani’s* peculiar bi-subunit DNA topoisomerase-IB and to have potent anti-leishmanial activity in the BALB/c mouse model of leishmaniasis [24]. This spiro chemical “8′-ethyl-4′-(4-hydroxy-3-methoxyphenyl)-3′-methyl-3′,4′-dihydro-1′H-spiro[indoline-3,2′-quinolin]-2-one” also known as JS87 was evaluated at different stages of the life cycle of *L. braziliensis* parasites. JS87 was able to inhibit the growth of promastigotes without affecting the viability of the mammalian cells and decrease the number of intracellular amastigotes of *L. braziliensis* [25]. JS87 was discovered to impact the sterol biosynthesis pathway at the SE enzyme level and modify internal parasite control by interrupting the regulatory volume decline. These findings show that the presence of the spiro ring structure of quinoline and oxindole scaffolds improves anti-leishmanial activity, potentially paving the way for the development of effective quinoline-oxindole hybrids against *L. braziliensis*, the main etiological agent of cutaneous leishmaniasis in South America. The orotate phosphoribosyltransferase (OPRT) domain catalyzes the creation of UMP from scratch. The cis-spirooxindoles also show a strong binding affinity for the OPRT domain of the *L. donovani* uridine 5′-monophosphate synthase (LdUMPS) [26]. However, extensive research is warranted to understand the exact mode of action of spirooxindoles against leishmaniasis.

Sterol 14a-demethylase is a mixed-function oxidase from the cytochrome P450 (CYP51) superfamily that metabolizes a variety of chemical substrates. Sterol 14a-demethylase catalyzes the oxidative removal of the 14a-methyl group from the sterol core, releasing two electrons and two protons [27]. The 14a-demethylation in *Leishmania* most likely happens after the first C4-demethylation. Furthermore, C24-methylation can occur in these parasites before and after 14a-demethylation. It appears that enzymes involved in the post-squalene sterol biosynthesis enzymes in *Leishmania*, such as sterol C4-demethylase, sterol 14a-demethylase, and sterol C24-methyltransferase, have enough flexibility to accept diverse substrates [28]. Inactivation of sterol 14a-demethylase in promastigotes causes membrane instability, increased membrane fluidity, lipid rafts failure, heat stress hypersensitivity, morphology alternations, and cytokinesis deficiency. 14a-demethylase is not essential for promastigote survival or proliferation in *L. major*, but it is important in cell shape, differentiation, and division [21]. Recently, an electronic assessment of the FDA-approved drug library against *L. donovani* sterol alpha-14 demethylase using a drug repurposing approach suggested that Avodart significantly reduced the number of intra-macrophagic amastigotes. Avodart-induced ROS caused apoptosis-like cell death in the parasites, which was identified by annexin V/PI staining [29].

The azole antifungals are a class of antifungal drugs that operate in the ergosterol biosynthesis pathway. They have a broad spectrum of activity against most yeasts and filamentous fungi [27]. Antifungal azoles work by inhibiting the enzyme sterol 14a-demethylase in *Leishmania* parasites [21]. Aryl Quinoline inhibits the sterol biosynthesis pathway in *L. braziliensis* promastigotes [30]. The mode of action includes disruption of parasite bioenergetics by disruption of mitochondrial electrochemical potential, alkalinization of acidocalcisomes, and suppression of the ergosterol biosynthetic pathway in promastigote forms [31]. The 6-ethyl-2-phenylquinoline inhibited the growth of *L. braziliensis* promastigotes on bone marrow-derived macrophages without compromising the viability of the mammalian cells and reduced the amount of intracellular *L. braziliensis* amastigotes [32]. In treated parasites, an increase in squalene and a decrease in 5-dehydroepisterol is seen. A similar effect is observed in promastigotes of *L. mexicana* exposed to miltefosine at the same time [33]. Altogether, these findings support the effectiveness of quinoline chemicals as leishmanicidal.

Statins are powerful inhibitors of cholesterol production; by inhibiting HMGR, a rate-limiting enzyme in sterol synthesis, statins stall cholesterol synthesis. Since the fate of *Leishmania* spp. infections are influenced by the host’s cholesterol levels [34] many protozoan parasites, such as *Plasmodium falciparum* and the *Trypanosomatid* family, including *T. cruzi* and various *Leishmania* spp., have been discovered to be inhibited by statins. The anti-leishmanial potential of inhibitors belonging to the statin group of compounds such as atorvastatin, simvastatin, and antidepressants such as ketanserin and mianserin [35,36] has been proven by targeting the HMGR enzyme. Mevastatin, also known as compactin or ML236B, is a polyketide medication that is used as an anti-cancer treatment, cholesterol-lowering pharmaceuticals, and immunosuppressants [17]. Exogenous ergosterol addition to these statins-treated parasites was able to overcome growth inhibition. However, cholesterol supplementation did not help the statin-inhibited promastigotes [35].

A geometrical fit was found when fenarimol (A comparatively innocuous herbicide, [alpha-(2-chlorophenyl)-alpha-(4-chlorophenyl)-5-pyrimidinemethanol],) was docked to the hypothesized catalytic binding site of *L. major’s* 14-lanosterol demethylase [37]. In silico analysis of fenarimol and catalytic binding site of *L. major’s* 14-lanosterol demethylase [37] revealed that fenarimol or its derivatives could be used to build anti-leishmanial medications. Licorice, or Glycyrrhiza glabra, is one of the crucial medicinal plants for treating respiratory disorders and inflammatory processes. The mode of action of glycorrhizic acid (GA) in *Leishmania* has been proposed via several mechanisms. These mechanisms include prostaglandin E2 (PGE2) inhibition and NO production [38]. It also reduced the expression of the multidrug resistance-associated protein1 (MRP1) gene and ABC transporter MRPA (PGPA) transporters [39]. In addition, other modes of action, such as inhibition of the HMGR enzyme and depleting ergosterol levels, which leads to parasite mortality [40], are also observed. However, GA has various mechanisms of action and multiple targets in the parasite, which have been widely studied.

### 2.2. Purine Salvage Pathway

**Figure 2:**
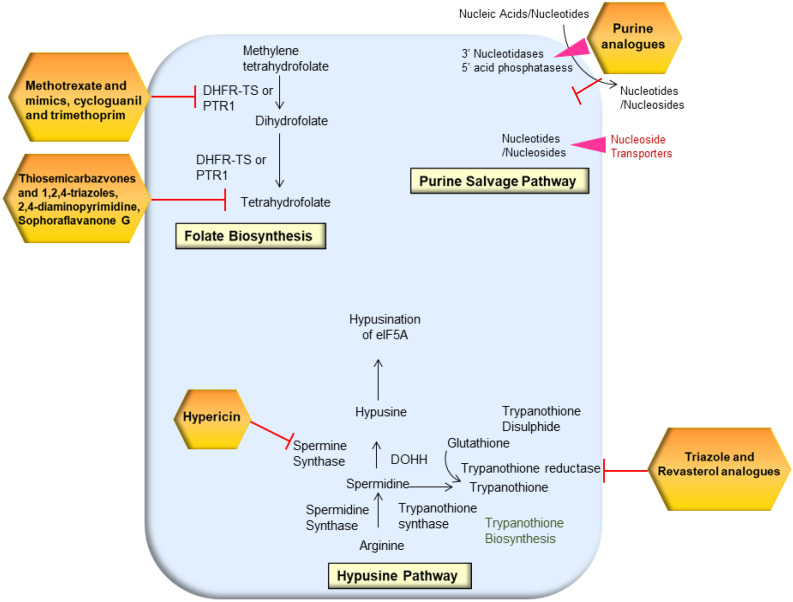
Targeting the folate, purine, and hypusine biosynthesis pathways in *Leishmania* through a drug repurposing approach.

Mammalian cells make purine nucleotides from amino acids and one-carbon moieties, whereas protozoan parasites cannot make purine nucleotides from scratch. Exogenous purines are funneled into hypoxanthine by *L. donovani* promastigotes. According to metabolic, biochemical, and genetic investigations, the enzyme HGPRT recycles purines inside parasitic cells, which is one of its most essential biocatalytic functions. Purines (adenine and guanine) are recovered by the salvage pathway from nucleotide metabolism degradation products as well as hypoxanthine and xanthine [41]. Multiple nucleoside hydrolase enzymes have been identified in *Leishmania* that catalyzes the degradation of nucleosides, nucleotides, and nucleic acids in the host before entering the parasite purine pools. The external cell surface of *L. donovani* expresses two membrane-bound 3′-nucleotidases/nucleases [42]. Thus, targeting the purine salvage pathway can be effective in limiting parasite growth and infection (Figure 2).

One of the effective techniques to target the purine salvage pathway is using the purine pyrimidine analogues. The purine pyrimidine analogues technique is centered on the creation of purine ribonucleoside prodrugs that are inactive in mammals but activate in parasites to produce harmful metabolites [43]. It has been discovered that nucleosides, particularly adenosine, have a strong influence on metacyclogenesis [44]. *Leishmania* parasites trigger the purine salvage pathway under purine stress by upregulating purine metabolic pathway enzymes and transporters [45]. In addition to it, pyrimidine analogues also have an antiproliferative impact. 5-fluorouracil, a pyrimidine nucleoside analogue, is transformed in vivo to the active metabolite 5-fluoroxyuridine monophosphate (F-UMP), which binds to RNA and inhibits cell development. The *Leishmania* parasites have the ability to biosynthesize and salvage pyrimidines from their host environment. However, *L. donovani* mutants that lack the pyrimidine salvage LdUPRT enzyme was hypersensitive to uracil, 5-fluorouracil, and 4-thiouracil at high doses [46]. Cytarabine and 5-fluorouracil were the most effective inhibitors against promastigotes, whereas azathioprine and 5-fluorouracil were comparatively more effective against the amastigote stage. The ultrastructural analysis revealed significant cytoplasmic vacuolization, mitochondrial enlargement, and the formation of autophagosome-like structures in the presence of azathioprine and 5-fluorouracil. 5-fluorouracil induced changes in the kinetoplast, finally resulting in an autolysis process and intracellular parasites’ mortality. The design and manufacture of a library of pyrazolo [3,4-d] pyrimidine nucleosides were disclosed, and their action against *T. cruzi* and *L. infantum* intracellular amastigotes was tested in vitro [47]. In human and animal liver microsomes, a 4-chlorophenyl substituent on the 7-position provided good anti-*T. cruzi* efficacy and selectivity while also being metabolically stable.

Nucleoside transporters 1–4 (NT1, NT2, NT3, and NT4) are four purine transfer sites found in the *Leishmania* genome. NT3 permease produces a considerable amount of mRNA during parasite growth and has a strong affinity for purine nucleobases [48]. Ortiz et al. suppressed the nucleobases of the nucleoside transporter 3 (NT3) permease using antisense RNA technology implicated in *Leishmania’s* salvage pathway to disrupt purine nucleotide uptake and, as a result, parasite death [48].

An investigation has found that glucose and/or amino sugars are required for the amastigote stage, albeit they may be primarily required for glycoconjugate formation [49]. *Leishmania* requires gluconeogenesis for virulence, according to a gene deletion study of the gluconeogenic enzyme fructose-1,6-bisphosphatase (FBP) [50]. In gluconeogenesis, GAPDH can also catalyze the reverse process from 3-phospho-D-glyceroyl phosphate to GAP. *L. donovani* cytosolic GAPDH (cGAPDH)-null mutant had lower infectivity in visceral organs [49,51]. When *L. donovani* is present as an amastigote in the macrophage phagolysosome, where sugar levels are low, cGAPDH is involved in glycolysis and/or gluconeogenesis pathways [49,51]. Moreover, the translocation of GAPDH to the nucleus in response to cellular stress is evidence of GAPDH involvement in nonmetabolic processes in higher eukaryotic cells [52]. When these *Leishmania* cells were exposed to cellular stress, such as serum withdrawal or stress generated by hydrogen peroxide exposure, no translocation of GFP-cGAPDH fusion proteins into the nucleus was observed. However, it is impossible to rule out the idea that *L. donovani* cGAPDH has functions other than glycolysis [51]. This implies that it would be ideal for creating cGAPDH [51] inhibitors in *L. donovani*. The ideal inhibitor, on the other hand, should be capable of inhibiting both glycosomal and cytosolic GAPDH activity. Recently, antimalarial drugs such as artesunate, quinine, and mefloquine showed significant anti-leishmanial activity by targeting parasites’ glycolytic enzymes, mainly GAPDH [53]. Moreover, an in silico study identified multiple approved drugs which can interact with glycolytic enzymes in the different forms of leishmaniasis except against VL [54]. Khanra et al. identified Suramin as an effective and efficient anti-leishmanial drug [55]. Suramin is being used to treat African trypanosomiasis (sleeping sickness) caused by *T. brucei*, and it has been shown to block the parasite’s glycolytic enzymes [56]. Moreover, Suramin has also shown promise as a potent anti-viral and anti-cancer agent [57]. Multiple enzymes of the glycolytic pathway such as enolase, and aldolase, are recognized as potential Th1-stimulatory proteins in PBMCs from *Leishmania*-infected hamsters and leishmaniasis patients [58,59]. Because the host T-cell responses are critical in managing VL, these *Leishmania* proteins that kindle the host T-cell response can be considered to be effective vaccine and drug targets [60,61].

### 2.3. Glycosyl Phosphatidyl Inositol (GPI) Biosynthesis

GPI biosynthesis is the process of adding sugars and ethanolamine to phosphatidylinositol (PI) in consecutive order [62]. GPI molecules help the parasites to survive and multiply in two hostile habitats and act as anchors for surface molecules such as proteins in the vector (*phlebotomus*) and the human host [63]. LPG, glycol inositol phosphor lipids (GIPLs), glycoproteins 63 (gp63), and the proteophosphoglycan (PPG) are all GPI-anchored components in *Leishmania*. They also comprise secreted phosphoglycans (PGs) containing proteins, such as the secretory-PPG (sPPG) and a secreted acid phosphatase (sAP) [64].

**Figure 3:**
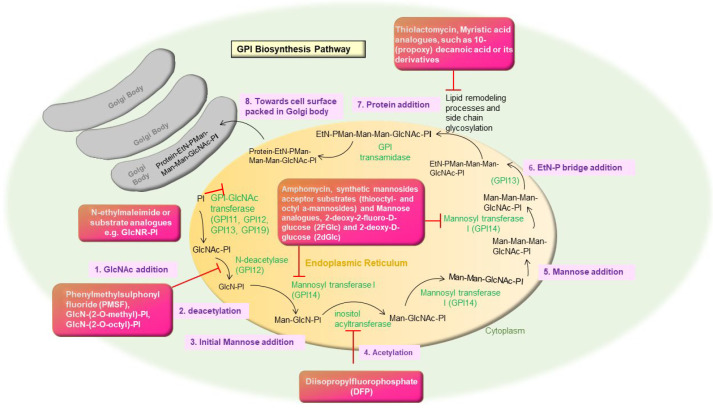
Inhibition of GPI biosynthesis pathway in *Leishmania* by different repurposed and/or potential drug candidates.

The enzymes involved in GPI biosynthesis are found to be inhibited by several suphydryl alkylating agents such as N-ethylmaleimide, substrate analogues such as GlcNR-PI, diastereoisomer with L-myo-inositol, the substitution of the 2-hydroxyl group of the D-myo-inositol with a methyl or octyl group, or GlcNAc-b-PI [65]. A serine esterase inhibitor phenylmethylsulphonyl fluoride (PMSF), GlcN-(2-O-methyl)-PI, GlcN-(2-O-octyl)-PI, and diisopropylfluorophosphate (DFP) inhibit inositol acylation and diacylation steps respectively [66]. Moreover, they cause significant disruption in the active equilibrium between mature acylated and non-acylated intermediates. In parasitic protozoa, the lipopeptide antibiotic amphomycin forms a compound with Dol-P (mannose donor) in the presence of Ca^2+^, limiting GPI production in vitro by blocking the interaction between the Dol-P-Man synthase and Dol-P [67]. In vivo, synthetic mannosides acceptor substrates (thiooctyl- and octyl a-mannosides) and Mannose analogues, 2-deoxy-2-fluoro-D-glucose (2FGlc) and 2-deoxy-D-glucose (2dGlc), have been shown to impede Dol-P-Man synthesis [68]. N-4-(-5(trifluromethyl)-1-methyl-1H benzo (d)imidazole-2 yl) phenyl) was docked to GPI14 in eight different ways. The interaction of GPI 14 with docked chemicals revealed that mannosylation was inhibited, implying druggability for leishmaniasis treatment [69] (Figure 3).

Moreover, the parasite could also be killed by inhibiting the fatty acid route using thiolactomycin, a fatty acid synthesis inhibitor [70]. Myristic acid analogues, such as 10-(propoxy) decanoic acid or its derivatives, were also found to be harmful to trypanosomatids but not to mammalian cells studies [71].

### 2.4. Folate Biosynthesis Pathway

Tetrahydrofolate functions as a one-carbon unit donor or acceptor in biosynthetic and degradative processes [72] and is required for the biosynthesis of purines, thymidylate, pantothenate, RNA, and amino acids, including methionine and glycine-to-serine conversion. Although eukaryotes can receive folate from the media, some steps of the folate pathway, such as folate regeneration through dihydrofolate reduction and the formation of glutamate tetrahydrofolate, are conserved [73]. The key co-factors tetrahydrofolate and decreased pterin are required in the metabolic pathways associated with the transfer of one-carbon units in *Leishmania* parasites [74]. DHFR is an important enzyme in folate metabolism and thymidine synthesis. (MTX, 1), cycloguanil and trimethoprim (TMP, 2) are therapeutically employed DHFR inhibitors that contain a variety of bioactive moieties. These two inhibitors, on the other hand, have exhibited poor selectivity for *L. donovani* DHFR [75]. Sharma et al., identified four compounds, ZINC57774418 (Z18), ZINC69844431 (Z31), ZINC71746025 (Z25), and D11596 (DB96), as new leads for LdDHFR inhibition based on stable interactions and binding free energy data. The study used methotrexate and LEAD (5-(3-(octyloxy)-benzyl) pyrimidine-2,4-diamine) to dock in the active site of the LdDHFR to comprehend the binding mode features of the substrates/inhibitors in the LdDHFR. These four hits display higher selectivity for LdDHFR, allowing them to be processed for further chemical and biological evaluation and optimization [76].

The 3,4-dihydropyrimidine-2-one and 5-(3,5-dimethoxybenzyl) pyrimidine-2,4-diamine motifs were employed to produce novel antifolates. The actions of DHFR and thymidylate synthase (TS) are effectively separated in humans. *Leishmania* has a bifunctional dihydrofolate reductase-thymidylate (DHFR-TS) in contrast to human cells [77]. C-5 and C-6 dihydropyrimidine (DHPM) derivatives have already been described as effective anti-leishmanial agents [78]. Pyrazole derivative was shown to be the most effective anti-leishmanial agent. Trimethoprim-based methotrexate mimics with oxoethyl linker displayed exceptional inhibition ability and selectivity for LmDHFR. However, further structural modifications in dihydropyrimidine 5-carboxamides and 5-benzylpyrimidine-2,4-diamine, imperative scaffolds to develop potential anti-leishmanial drugs are warranted [79].

*Leishmania* species are resistant to the majority of DHFR-TS inhibitors [80]. When DHFR-TS is blocked, an alternate salvage mechanism mediated by PTR1 provides ample folate supply [81] while PTR1 is not found in mammalian hosts. In trypanosomatids, PTR1 is an NADPH-dependent short-chain reductase. *Leishmania* parasites are known to salvage pteridines from their hosts, PTR1 aids in the conversion of biopterin to tetrahydrobiopterin, which is incorporated by *Leishmania* from the host cell. In the absence of DHFR-TS, PTR1 also has a proclivity for converting folates to tetrahydrofolates. Therefore, inhibiting both DHFR-TS and PTR1 at the same time is required to target folate production in leishmanial cells [77,82].

Some thiosemicarbazones and 1,2,4-triazoles have been reported to be inhibitors of DHFR and PTR1, respectively [83,84]. As an anti-leishmanial agent, Temraz et al., hypothesized this novel 1,4-disubstituted-1,2,3-triazole/thiosemicarbazone framework that includes coupling the thiosemicarbazone pharmacophore with a click-modifiable triazole on bifunctional aromatic skeletons such as vanillin, isovanillin, and 4-hydroxyacetophenone would result in a promising anti-leishmanial candidate [85]. The unique propargyl group pharmacophore and among the 4-hydroxyacetophenone-derived triazoles, the 4-bromophenacyl substituted triazole had the highest activity, against promastigote and amastigote. In another study, Benzimidazole and benzoxazole-based dual inhibitors of DHFR-TS and PTR1 were developed using a structure-based drug design. 2-(4-((2,4-dichlorobenzyl)oxy)phenyl)-1H-benzo[d]imidazole and 2-(4-((2,4-dichlorobenzyl)oxy)phenyl)-1H-benzo[d]imidazole -1H-benzo[d]oxazole molecules were moderately active, with IC50 values of 68 ± 2.8 µM and 57 ± 4.2 µM, respectively [86]. Virtual screening, docking, and molecular dynamic simulations were used to validate both structure-based models, which were then used to create dual inhibitors of DHFR-TS and PTR1. After that, the compounds were produced, described, and tested for anti-leishmanial activity in vitro. Hermann et al. identified 15 natural products with a significant anti-leishmanial activity using in silico and in vitro methods. Among them, Sophoraflavanone G was found to be the most potent [87]. However, further research is warranted for rational drug design.

The 2,4-diaminopyrimidine derivatives replaced at the sixth position are found to be competitive inhibitors of both enzymes, but the 2,4-diaminoquinazoline derivative is a discerning inhibitor of *L. chagasi* pteridine reductase 1 (LcPTR1) [88]. This knowledge will aid in the development of powerful inhibitors with varying selectivity profiles. Quinazolin-4(3H)-ones with attachments at the 2- and 3-positions of the quinazoline core have recently received a lot of attention as an anti-leishamanial agent [89], and some of their derivatives, such as 1,2-substituted quinazolin-4(3H)-ones and 2-aryldihydroquinozalinones, have shown discrete to excellent anti-leishmanial activities with one-digit micromolar IC50 on proliferative promastigote or amastigote of *Leishmania* parasite [90,91]. Romero et al. reported the anti-leishmanial activity of a series of 2-(substituted-aryl)-quinazolin-4(3H)-ones, with no containing substitution at the 3-position, against different *Leishmania* species responsible for American cutaneous leishmaniasis: *L. braziliensis*, *L. Mexicana*, and *L. amazonensis*, based on the important role of quinazoline scaffold in the design of novel and potent anti-leishmanial [92]. The methoxy substitution at position two or three on the Y aryl ring and the chlorine atom at position six on the quinazolin fused ring appears to be crucial in the biological activity of these 2-arylquinazolin-4(3H) ones, according to structure-activity relationship research.

### 2.5. Trypanothione Pathway

Because oxidative stress is so important in the host’s immunological response to infection, parasite survival is largely dependent on the parasite’s capacity to withstand it. In infectious trypanosomatids, TSH2, being the principal redox reactive metabolite, participates in a variety of enzymatic and non-enzymatic activities by shuttling electrons to numerous acceptors involved in important parasite survival mechanisms such as peroxide detoxification and DNA synthesis [93,94]. These parasites lack catalase and other redox-controlling mechanisms; thus, they rely on TSH2, a unique glutathione variant that plays a key role in thiol homeostasis [93,95]. Because of its importance in trypanosomatids and its lack in humans, the TSH2 pathway presents a unique opportunity for the discovery of medications that can prevent protozoan survival while causing no harm to the host [96]. Furthermore, because TSH2 metabolism is a unique mechanism shared by all trypanosomatids, targeting key enzymes in this pathway could help treat a variety of infections [97].

TSH2 is a vulnerability for these parasites because it is both important and unusual, and all related enzymes are regarded as promising treatment candidates [98]. TSH2 is made in two ATP-dependent steps, each of which involves the conjugation of spermidine (Spd) with two glutathione (GSH) molecules. Spd binds to the glycine carboxyl moiety of GSH to form a glutathionyl Spd intermediate (Gsp), which then connects with another molecule of GSH to form TSH2. TSH2 is involved in a variety of tasks, including xenobiotic detoxification, DNA synthesis, and oxidant defense, by delivering reducing equivalents to various molecular partners (e.g., Tryparedoxin, TXN). At the expense of NADPH, trypanothione reductase (TR) regenerates TSH2 from trypanothione disulfide (TS2) [94]. TR, the enzyme directly responsible for retaining TSH2 in the reduced state, has been investigated intensively since it meets the majority of the criteria for a suitable therapeutic target [98]. Saccoliti et al. recognized thioether derivative RDS 777 (6-(sec-butoxy)-2-((3-chlorophenyl)thio)pyrimidin-4-amine) (IC501 429.43 mM), which binds to the catalytic site of TR and forms hydrogen bonds with the catalytic residues Glu466’, Cys57, and Cys52, limiting TSH2 binding and preventing its reduction [99]. Similarly, ammonium trichloro [1,2-ethanediolato-O,O′]-tellurate (AS101), a tellurium-based non-toxic immunomodulator, forms thiol bonds with cysteine residues of TR in *Leishmania* promastigotes resulting in the parasite’s inactivation and induction of ROS-mediated apoptosis via increased Ca^2+^ levels, loss of ATP, and mitochondrial membrane potential, as well as metacaspase activation [100]. AS101 can block integrin-dependent PI3K/Akt signaling and activate host MAPKs, and nuclear factor (NF)-κB in *L. donovani* infected macrophages, inhibiting the interleukin (IL-10)/STAT3 pathway and activating host MAPKs and NF-κB for Th1 response.

Disrupting the homodimeric interface of *L. infantum* trypanothione disulfide reductase (LiTR) has proven to be an alternative and underutilized technique in the hunt for novel anti-leishmanial drugs. The pyrrolopyrimidine and 5-6-5 imidazole-phenyl-thiazole -helix-mimetic scaffolds were decorated with substituents that could imitate three essential residues (K, Q, and I) of the linear peptide prototype using a proteomimetic technique (PKIIQSVGIS-Nle-K-Nle) [101]. This allowed the blocking of the enzyme as well as the killing of external and intracellular parasites. In comparison to reference imidazoles, certain compounds with (poly) aromatic substituents significantly improve the capacity to disrupt LiTR dimerization. Revuelto et al. recently revealed symmetrical triazole analogues with a much more potent inhibitory effect was able to kill both external and intracellular parasites [102]. The anti-leishmanial action of various resveratrol analogues (ResAn) was investigated utilizing molecular modeling, computational docking, and in vitro research, with a focus on their pro-oxidant effect. ResAn may be a viable medication candidate with properties to traverse biological membranes and high gastrointestinal absorption, not violating Lipinski’s criteria. ResAn’s anti-leishmanial impact has been linked to a pro-oxidant effect which can be subjugated as an antimicrobial agent [103]. Virtual evaluation of 600,000 ZINC compounds yielded a selection of 20 drug-like molecules with high affinity for LiTR (docking scores of 11.6 kcal/mol to 12.7 kcal/mol) [104]. Molecular dynamics analysis revealed an N-(6-quinolinemethyl)-3-pyrazole carboxamide and the involvement of the quinoline nucleus in the hydrogen bond formation with the active site of LmTR. As a result, this molecule offers a good starting point in our hunt for more effective and less cytotoxic anti-*L. mexicana* medicines [105]. According to computer simulations, aminoguanidine hydrazones (AGH) and thiosemicarbazones (TSC) showed satisfactory anti-leishmanial efficacy. These hits are capable of blocking TR in amastigote forms [106]. In another study, the molecular docking results have revealed a preferential binding profile for Masticadienonic acid and 3-Methoxycarpachromene ligands inside the active region of TR. According to the ADMET study, the 3-Methoxycarpachromene has the best chance of being absorbed by humans [107].

The chloroform extract of *Corchorus capsulari* leaves significantly inhibits the *L. donovani* promastigotes. In promastigotes, β-Sitosterol CCL disrupts the redox balance via intracellular ROS production, triggering a variety of apoptotic events such as structural changes, increased lipid body storage, mitochondrial membrane depolarization, phosphatidylserine externalization, and non-protein thiol depletion. In addition, enzyme inhibition and an in silico investigation revealed that β-sitosterolCCL inhibits *L. donovani* TR, indicating that it has anti-leishmanial activity (LdTR). Overall, β-sitosterol CCL looks to be a novel LdTR inhibitor and could be a promising therapy option for VL in the future [108]. The crude methanolic extract, n-hexane, and n-butanol fractions of the seeds of the plant *Abutilon indicum* showed significant efficacy against *Leishmania* promastigotes and intracellular amastigotes survival in hamsters [109]. In another study, Inacio et al. investigated (-)-Epigallocatechin 3-O-gallate (EGCG), a flavonoid molecule found in green tea leaves, and its leishmanicidal mode of action on *L. infantum* promastigote growth in a concentration-dependent manner [110]. EGCG binds to TR and acts as a competitive inhibitor of the TSH2 substrate.

In addition to the above-mentioned targets, enzymes such as arginase (ARG), ornithine decarboxylase (ODC), S-adenosylmethionine decarboxylase (AdoMetDC), Spd synthase (SpdS), trypanothione synthetase (TryS or TSA), TR, and tryparedoxin peroxidase (TXNPx) are responsible for the Spd metabolism. Therefore, these enzymes are potential targets for the advancement of new drug development against leishmaniasis [16,111].

### 2.6. Hypusine Pathway

The unusual hypusine amino acid is present in two eukaryotic proteins, eukaryotic translation initiation factor (eIF)-5A1, and eIF5A2. Hypusine is attached to the lysine residue of these proteins through a post-translational modification known as hypusination. This is aided by two enzymes, namely-deoxyhypusine synthase (DHPS) and deoxyhypusine hydroxylase (DOHH), that utilize the polyamine spermidine substrate and form hypusinated eIF5A [112].

The structural differences between the human homolog and *L. donovani* DOHH (LdDOHH) could be used to build selective inhibitors against the parasite using a structure-based design [113]. LdDOHH activity was reduced to 14% of wild-type recombinant LdDOHH activity after deletion of the ten-amino-acid-long insertion. In vitro, metal chelators such as ciclopirox olamine (CPX) and mimosine dramatically reduced *L. donovani* growth and DOHH activity [114]. The parasite enzyme was more resistant to these inhibitors than the human enzyme. Trypanosomatids contain two eIF5A isotypes and two deoxyhypusine synthase (DHS) enzyme isotypes that are responsible for their post-translational modification. Only DHS1 is catalytically active, although both eIF5A isotypes are functional. Small antisense RNA-mediated gene silencing revealed that both DHS1 and two genes were necessary for *E. histolytica* trophozoite proliferation in vitro. Only the eIF5A2 gene was actively transcribed in trophozoites, but not the eIF5A1 gene. Silencing of the eIF5A2 gene resulted in compensatory upregulation of the eIF5A1 gene, implying that the two eIF5A isotypes have similar roles and emphasizing the necessity of eIF5As for parasites proliferation and survival. The findings highlight the importance of eIF5A and DHS for parasite proliferation and maybe differentiation and suggest that the hypusination-associated pathway could be a new reasonable target for treatment development. When DHS1 and DHS2 were co-expressed in *E. coli*, DHS activity rose 3000-fold, indicating that the formation of a heteromeric complex is required for maximal enzymatic activity [115].

Arginase, the first enzyme in *Leishmania*’s polyamine biosynthesis pathway, has been identified as a possible therapeutic target. Both macrophages and amastigotes need a lot of arginines, which is a metabolic bottleneck that determines the fate of *Leishmania* infection. *Leishmania* parasites obtain arginine through a mono-specific, high-affinity transporter (amino acid permease 3, AAP3) that transports arginine into *Leishmania* cells [116]. The flavonols quercetin and fisetin, as well as green tea flavanols such as catechin (C), epicatechin (EC), epicatechin gallate (ECG), and epigallocatechin-3-gallate (EGCG), and cinnamic acid derivates such as caffeic acid, limits the parasite enzyme and modulate the host’s immune response to parasite defense, with lesser toxicity to the host [117]. Arginine is the sole source of Spd formation and, as a result, trypanothione biosynthesis in *Leishmania*. Arginine is also the source of hypusine synthesis, a unique precursor for eIF5A1, via Spd [118]. *L. donovani* promastigotes activate arginine transport in response to amino acid deficiency, as evidenced by a four-fold rise in lipid droplet-associated proteins (LdAP3.2) mRNA (LinJ. 31.0910) and protein levels [119]. Castilho-Martins et al. discovered that the *L. amazonensis* homolog of LdAAP3 had a comparable response according to external and internal sensing mechanisms [120]. The rate of arginine transport is further affected by the activity of the polyamine pathway, for which arginine is the sole precursor. *L. donovani* mutants missing either ornithine decarboxylase or Spd synthase take up half as much arginine as wild-type parasites, and their cellular arginine pool is correspondingly reduced. This shows that around half of the arginine collected in promastigotes is directed to polyamine production and that this pathway regulates arginine transport activity [116]. The hypericin treatment caused the *Leishmania* parasite to become spermidine-depleted and die (Figure 2). The parasite’s increased intracellular ATP and NAD^+^ levels, as well as its decreased histone type III histone deacetylase (Sir2RP) expression, are cytoprotective mechanisms against ROS generated by hypericin treatment, possibly by inducing autophagy [121].

### 2.7. Other Pathways of Leishmania Parasite Targeted by Various Drug Candidates

In addition to the compounds targeting the major metabolic pathways (discussed above) of the parasite, several other drugs have been reported to limit the proliferation and survival of *Leishmania*.

Delamanid (OPC-67683) is an anti-tuberculosis drug that was tested for anti-parasitic properties both in vitro and in vivo. Delamanid is quickly metabolized by parasites via nitroreductase enzyme that activates fexinidazole. Delamanid has shown promising results for the treatment of *Leishmania* infections, and they raise the possibility of targeting other protozoan infections whose pathogenicity is similar to *Leishmania* [122] Delamanid was found to be more effective than miltefosine, with no overt evidence of toxicity in the mice, demonstrating delamanid’s therapeutic potential [122]. However, the precise identification of the target and the metabolites it generates is still being researched, which makes it difficult to predict the result of delamanid treatment in VL patients. In vitro anti-leishmanial activity against intracellular amastigotes was also discovered in close congeners of naturally occurring b-nitrostyrenes [123]. Even at a dosage of 25 µM, these chemicals were shown to be harmless to mammalian macrophages. It is obvious from the data that the alkyl substitution at the b position has a significant impact on the biological activity against *L. donovani* promastigotes and amastigotes. A variety of dihydropyrimidine-based derivatives have recently been created to evaluate their efficacy as anti-leishmanial compounds by forming particular contacts in the active site of pteridine reductase 1 (PTR1) in *L. major* [124].

The biological activity of Copper complexes with fluorinated β-diketones was tested against *L. amazonensis* [125]. Following that, several other synthetic compounds, including the S2 complex (an organic complex of copper chloride, ascorbic acid, and nicotinamide) [126], acetylsalicylic acid [127], and immunomodulatory peptide from cystatin [128], were shown to have both immune modulating and anti-leishmanial properties. Curdlan is a b-(1, 3) glucan with immunomodulatory and pharmacological properties and according to a study [129]. Curdlan, at a dose of 10 mg/kg/day, virtually totally eradicated the parasite burden in the liver and spleen in an experimental BALB/c mouse model of VL due to the generation of disease-resolving Th1 and Th17 cytokines such as IL-17 and IL-23 respectively [3]. Glycyrrhetinic acid (GRA), a compound derived from the root of the medicinal plant licorice (*Glycyrrhizza glabra* L.), has been shown to produce dominant Th1 immunity, and it blocks prostaglandin-E2 synthesis via blockade of cyclooxygenase- (COX-) 2 resulting in concurrent augmentation nitric oxide production in Leishmania-infected macrophages through the enhancement of iNOS2 mRNA secretion [37]. GRA has potent anti-leishmanial activity, as demonstrated by in vivo administration of GRA in a BALB/c mouse model of VL, which almost eliminated parasite burden in the liver and spleen. NO upregulation, proinflammatory cytokine expression, and nuclear factor-κB (NF-κB) activation were found to be involved in GRA’s anti-leishmanial effect [38]. In *L. donovani*-infected mice, an adipocyte-derived hormone called leptin was discovered to be capable of regulating the immune response. The treatment of splenocytes with recombinant leptin reduced parasite burden by increasing NO and proinflammatory cytokine production, indicating a host-protecting Th1 response [130]. Tannins and similar chemicals have also been shown to kill *Leishmania* parasites through NO [131]. IL-12 upregulation has been identified in a variety of plant secondary metabolites and extracts, showing the usefulness of various natural resources as anti-leishmanial medications. IL-18, which, along with IL-12, stimulates interferon (IFN)-γ production and aids in parasite clearance, was also produced by stimulated macrophages. IL-18 mRNA was found to be induced by *Pelargonium sidoides* extracts during infection with *L. major* [132]. The levels of IL-6 and IL-10 cytokines were reduced in *L. major*-infected macrophages treated with Licarin A [133].

6-Nitro-2,3-dihydroimidazo [2,1-b] [1,3] oxazole derivatives were first investigated for tuberculosis as a backup to the clinical trial drug pretomanid (PA-824). It is also being considered a first-in-class medication candidate for VL. The phenyl rings of this compound were replaced by pyridine and arylpiperazine, and arylpiperidine as bioisosteres for a biaryl moiety to improve in vivo efficacy and aqueous solubility while preserving or enhancing potency against VL [134]. GSK3494245/DDD01305143/compound 8 is a small chemical that has been selected as a preclinical contender after demonstrating clinical-level effectiveness in a mouse model of VL. Compound 8 is a powerful and specific inhibitor of the parasite proteasome’s chymotrypsin-like activity. Compound 8 is nearing human clinical trials, raising optimism for better treatment options for this condition [135]. SQ109, a drug in phase IIb/III clinical trials to treat resistant *Mycobacterium* TB, has shown to be effective against Chagas’ disease-causing *T. cruzi* and cutaneous and mucocutaneous leishmaniasis-causing *L. mexicana*. The postulated mechanism of action on *L. mexicana* includes the collapse of the mitochondrial electrochemical potential (m), which disrupts the parasite’s internal Ca^2+^ homeostasis. SQ109, in addition to collapsing the parasite’s cilia, caused rapid damage to the parasite’s acidocalcisomes, which are critical organelles involved in bioenergetics and a variety of other functions, including Ca2^+^ homeostasis. Both of the drug’s effects on these organelles resulted in a significant increase in intracellular Ca^2+^ content, resulting in parasite death [136]. Likewise, amiodarone (AMD), a powerful antiarrhythmic, showed significant anti-leishmanicidal activity against *L. infantum* in vitro and in vivo [137].

Liposome formulations are very successful in the treatment of leishmaniasis and fungal infections [138]. Previous research has shown that adding phosphatidylserine to liposome membranes causes infected macrophages to target through the scavenger receptors [138,139]. The use of phosphatidylserine-treated liposomes has shown promise in the treatment of VL, enhancing antimony and furazolidone selectivity and therapeutic index in vitro [140]. Cinnarizine was examined in vivo as a free formulation and as a phosphatidylserine-liposome entrapment. It is the first antihistaminic medication with anti-leishmanial activity, and it could be utilized as a starting point for VL treatment development. When compared to the untreated group, the cinnarizine liposomal formulation reduced parasite burden in the liver by 54 percent; however, there was no change in the spleen of infected hamsters. Higher doses, new regimens, and delivery routes could be utilized instead to improve in vivo efficacy of cinnarizine [141]. Buparvaquone (BPQ), a hydroxynaphthoquinone sold under the brand name Butalex^®^ (MSD Animal Health Egypt), is a safe medication used to treat theileriosis in cattle [142]. In *Leishmania*-infected macrophages, BPQ upregulates some cytokines such as tumor necrosis factor, monocyte chemoattractant protein-1 (MCP-1), IL-10, and IL-6. This upregulation eradicates the parasites via a nitric oxide-independent method and targets Leishmania-infected macrophages of the spleen, liver, and bone marrow [143]. Sertraline [SRT; (1S,4S)-N-methyl-4-(3,4-dichlorophenyl)-1,2,3,4-tetrahydro-1-naphthylamine] is a selective serotonin reuptake inhibitor (SSRI) with acceptable side-effect and tolerance profiles for depression and anxiety disorders [144]. Sertraline promoted respiration uncoupling, a considerable drop in oxidative stress, and intracellular ATP level in *L. infantum* promastigotes in animal models against *Leishmania* infection [145]. The sertraline-induced metabolic disorder was shown by metabolomics, which revealed a wide range of polyamine biosynthetic intermediate and thiol-redox levels, as well as a scarcity of intracellular amino acids needed as metabolic fuel for *Leishmania* [146]. When entrapped in negatively charged liposomes (58 mV) phosphatidylserine liposomes (LP-SERT) of 125 nm, it drastically reduced parasite load in the mouse liver by 89 percent at 1 mg/kg while also lowering blood levels of IL-6 and increasing levels of the chemokine MCP-1 [147].

The anti-leishmanial activity of cardenolide digitoxigenin (DIGI) from a methanolic extract of *Digitalis lanata* leaves was assessed against *L. infantum* species. DIGI’s mode of action, according to preliminary research, was by modifying the mitochondrial membrane potential, increasing reactive oxygen species levels, and causing the parasites to accumulate lipid bodies. DIGI was integrated into Pluronic^®^ F127 (Sigma-Aldrich, Burlington, MA, USA) based polymeric micelles, and the resulting formula (DIGI/Mic) was utilized to treat *L. infantum*–infected mice. When compared to miltefosine- and DIGI-treated mice, DIGI/Mic generated better immunological responses [148]. Acarbose (ACA), a particular inhibitor of glucosidase-like proteins that have been used to treat diabetes, was found to be an anti-leishmanial agent in a comparable investigation. Furthermore, ACA combined with a Pluronic^®^ F127 (Sigma-Aldrich, Burlington, MA, USA) based polymeric micelle system dubbed ACA/Mic was found to be beneficial in treating *L. infantum*-infected BALB/c mice. Anti-leishmanial Th1-type humoral and cellular responses based on high levels of IFN, IL-12, tumor necrosis factor (TNF), granulocyte-macrophage colony-stimulating factor (GM-CSF), nitrite, and IgG2a isotype antibodies can be used to measure the drug’s leishmanicidal activity. In addition, the organ toxicity of ACA or ACA-treated animals was modest [149]. Buparvaquone is a promising leishmaniasis medication, although it has low bioavailability and therapeutic efficacy due to its hydrophobic nature. The parasite burden was nearly completely decreased by intraperitoneally administered buparvaquone-loaded (biogenic silicon nanoparticles) silicon nanoparticles made from barley husk, an agricultural residue that is commonly available [150].

## 3. Discussion

If left untreated, VL is a life-threatening illness. Although substantial research has been conducted in the hopes of discovering new leishmaniasis drugs due to the disease’s prevalence and severity, prominent pharmaceutical corporations have shown little interest in the subject. Here in this review, we have looked at the vital metabolic pathways in the *Leishmania* parasite to identify drugs as potent candidates or repurposed to treat leishmaniasis. There are analogous pathways, and enzymes identified that play a crucial role in parasites’ growth and proliferation but are absent in mammalian counterparts. These pathways and enzymes are precisely targeted via drug repurposing to treat leishmanial infection (Table 1) [21].

Studies reported that targeting enzymes involved in leishmanial sterol biosynthesis could significantly decrease parasite survival in the host [17,25,29]. Antimalarial drugs such as Spiro-indolone and spiro oxiindoles (JS87) target the enzyme SEs [25]. In contrast, antifungal azoles and aryl quinolines work at the level of sterol-14α-demethylase [27,28,32]. Another strategy that can be used to reduce the parasites’ survival is to lower the host’s cholesterol levels which severely affect the parasite’s proliferation [35,36]. Multiple cholesterol-lowering and antidepressant drugs such as statins have been repurposed to treat leishmaniasis, for instance, mevastatin, ketanserin, mianserin, atorvastatin, simvastatin, etc. [17,35,36]. However, further research studies are warranted for the wide use of these drugs against leishmanial infection. Purine-pyrimidine analogues are a potent method to deprive the pathogen of the purine nucleotides and thus, reduce its survival in the host [43]. In addition to that, there are enzymes discovered and studied at a different level of the purine salvage pathway, which could be targeted to inhibit *L. donovani*’s growth [47,151]. However, no drug repurposing studies have been conducted yet to target these enzymes. Similarly, GAPDH being the central enzyme to the glycolytic pathway in *L. donovani*, could be targeted for drug repurposing. Multiple enzymes of the glycolytic pathway, such as aldolase, enolase, triose phosphate isomerase, etc., are being targeted to develop vaccines against VL [61,152]. Still, these studies warrant more clinical data. In addition to that, there is extensive literature available on how GPI molecules help the pathogen evade the host’s immune response and promote infectivity [153,154,155]. This infers that these anchor molecules could serve as excellent drug targets against leishmaniasis. However, drug candidates against GPI biosynthesis and its intermediates still need to be explored.

*Leishmania* pathogen is unable to produce folate on its own, and folate is a nutritional requirement. DHFR is a critical enzyme in the metabolism of folate and, as a result, the bioavailability of thymine [74]. Pyrazoles, pyrimidine analogues, Methotrexate and its mimics, cycloguanil, trimethoprim, etc., are among the medications being explored to target DHFR in leishmaniasis [76,79]. Although inhibiting both DHFR-TS and PTR1 at the same time is required to target folate production in leishmanial cells [77,82]. Thiosemicarbazones and 1,2,4-triazoles, Benzimidazole and benzoxazole, Sophoraflavanone G, 2,4 diaminopyrimidine derivatives, and Quinazolin-4(3H)-ones are some of the drugs which are studied to be repurposed against VL [86,88,92]. Since TSH2 is both crucial and uncommon, all TSH2-related enzymes and pathways are considered attractive therapeutic possibilities for drug repurposing against VL [97,98]. Arginine is the precursor of Spd, which itself is the precursor of TSH2 [116]. Thus, producing leishmanicidal medications, agents that impact arginine transport into *Leishmania* cells, block polyamine production, trypanothione reduction, and elF5A hypusination should be intensively explored to limit the parasite survival [117,156,157]. Drugs such as thioether derivative, immunomodulator, pyrrolopyrimidine, and 5-6-5 imidazole-phenyl-thiazole -helix-mimetic scaffolds, resveratrol analogues, aminoguanidine hydrazones (AGH) and thiosemicarbazones (TSC), Masticadienonic acid, and 3-Methoxycarpachromene are being repurposed to target these enzymes [102,105,108,110]. Apart from repurposed drugs, multiple herbal extracts are being discovered to be utilized against *Leishmania* infection, including *Corchorus capsularis*, *Candida albican*, *Pelargonium sidoides*, *Glycorhyza glabra*, *Piper betle*, *Tinospora sinensis*, etc. [109,110,158]. Moreover, to increase the efficacy of repurposed drugs, nanoencapsulation and liposomal carriers are extensively being studied [142,147].

Apart from targeting the major metabolic pathways, there are various drugs reported to limit *Leishmania* infection by different mechanisms, such as using the adenosine analogues [159], anti-tuberculosis drug delamanid [122], dihydropyrimidine-based derivatives [124], TLR7 agonist [125], and curdlan [3].

Identification of novel drug targets can be beneficial in promoting the pipeline of disease elimination. In the current review article, we have discussed current and emerging drug targets in *Leishmania* emphasizing the crucial metabolic pathways of the parasite. The pursuit of novel drug targets sustained and resulted in the study of different metabolic pathways of *Leishmania* as the target for different drug repurposing approaches. Because of the high cost and time constrain of drug development, repurposing existing drugs to treat other diseases is becoming effective. Repurposing old drugs against leishmaniasis will potentially decrease the development cost and time constrain associated with drug discovery. Altogether, these factors would cumulatively help to reduce the mortality and morbidity associated with leishmaniasis.

## 4. Conclusions

In summary, targeting the enzymes involved in major metabolic pathways of the *Leishmania* parasite could significantly decrease parasite survival in the host. Repurposing the existing drugs against different metabolic pathways of the *Leishmania* parasite could significantly affect pathogen survival. Since the parasite cannot synthesize purines de novo, purine and pyrimidine analogues that turn toxic in the pathogen are other excellent candidates for leishmaniasis treatment. In addition, antifolates drugs that target DHFR-TS and PTR1 are widely being studied to counter leishmaniasis infection. Moreover, TR is the major redox controlling enzyme in *Leishmania* species, which is an excellent target to treat the infection. Inhibiting spermidine availability in the pathogen could hinder polyamine biosynthesis, hypusination of elF5A, and other major biosynthetic processes in the pathogen resulting in cell death. Thus, combining biochemical/metabolic target information and knowledge of drug repurposing novel drugs or combination therapies could be developed to block the infection and transmission of *Leishmania*.

## Figures and Tables

**Table 1:** Potential drug targets, repurposed drugs, and their mode of action to limit leishmaniasis.

*Leishmania* Species	Pathway	Drug Target	Potent Drug Candidate	Mode of Action	Reference
*L. braziliensis*	Sterol Biosynthetic Pathway	Squalene epoxidase	JS87	annulation of quinoline and oxindole scaffolds	[25]
*L. infantum*	Sterol Biosynthetic Pathway	Squalene epoxidase	spiro[cyclohexanone-oxindoles]	Inhibition of phosphodiesterase and tyrosine kinase	[23]
*L. donovani*	Sterol Biosynthetic Pathway	Squalene epoxidase	spiro[indole-3,3′-pyrrolizidine]-2-one	inhibitor of bisubunit DNA topoisomerase IB	[24]
*L. braziliensis*	Sterol Biosynthetic Pathway	sterol-14-α-demethylase	6-ethyl-2-phenylquinoline	disruption of mitochondrial electrochemical potential and alkalinization of acidocalcisomes	[32]
*L. donovani*	Sterol Biosynthetic Pathway	HMGR enzyme	Mevastatin	Inhibits HMGR activity	[17]
*L. donovani*	Sterol Biosynthetic Pathway	Sterol alpha-14 demethylase	Avodart	Avodart-induced ROS caused apoptosis-like cell death in the parasites	[29]
*L. major*	Sterol Biosynthetic Pathway	14-lanosterol demethylase	fenarimol	destabilization of membrane structure by inhibiting 14α sterol demethylase.	[37]
*L. donovani*	Sterol Biosynthetic Pathway	HMGR enzyme	Glycyrrhizic acid	inhibiting the HMGR enzyme	[40]
*L. donovani*	Purine Salvage Pathway	mRNA translation	5-fluorouracil 4-thiouracil	binds to RNA and inhibits cell development	[46]
*L. infantum*	Purine Salvage Pathway	mRNA translation	pyrazolo [3,4-d] pyrimidine	binds to RNA and inhibits cell development	[47]
*L. donovani*	Glycolytic Pathway	GAPDH	artesunate	targeting parasites’ glycolytic enzymes mainly Glycerol-3-phosphate dehydrogenase	[53]
*L. donovani*	Glycolytic Pathway	GAPDH	quinine	targeting parasites’ glycolytic enzymes mainly Glycerol-3-phosphate dehydrogenase	[53]
*L. donovani*	Glycolytic Pathway	GAPDH	mefloquine	targeting parasites’ glycolytic enzymes mainly Glycerol-3-phosphate dehydrogenase	[53]
*L. major*	Glycolytic Pathway	phosphoglycerate kinase	Suramin	inhibition of cytosolic phosphoglycerate kinase from	[55]
*L. major*	Glycosyl phosphatidyl inositol	mannosyltransferase	N-4-(-5(trifluromethyl)-1-methyl-1H benzo[d]imidazole-2 yl) phenyl)	inhibit mannosylation of glycosyl phosphatidyl Inositol	[69]
*L. donovani*	Folate Biosynthesis Pathway	DHFR	Methotrexate (MTX, 1),	Inhibit DHFR	[75]
*L. donovani*	Folate Biosynthesis Pathway	DHFR	cycloguanil	Inhibit DHFR	[75]
*L. donovani*	Folate Biosynthesis Pathway	DHFR	trimethoprim (TMP, 2)	Inhibit DHFR	[75]
*L. donovani*	Folate Biosynthesis Pathway	DHFR	ZINC57774418 (Z18)	Inhibits DHFR activity	[76]
*L. donovani*	Folate Biosynthesis Pathway	DHFR	ZINC69844431 (Z31)	Inhibits DHFR activity	[76]
*L. donovani*	Folate Biosynthesis Pathway	DHFR	ZINC71746025 (Z25)	Inhibits DHFR activity	[76]
*L. donovani*	Folate Biosynthesis Pathway	DHFR	and D11596 (DB96)	Inhibits DHFR activity	[76]
*L. donovani*	Folate Biosynthesis Pathway	DHFR	3,4-dihydropyrimidine-2-one	Inhibits DHFR activity	[77]
*L. donovani*	Folate Biosynthesis Pathway	DHFR	5-(3,5-dimethoxybenzyl) pyrimidine-2,4-diamine	Inhibits DHFR activity	[77]
*L. major*	Folate Biosynthesis Pathway	PTR1	thiosemicarbazones and 1,2,4-triazoles	Inhibit DHFR and PTR activity	[85]
*L. donovani*	Folate Biosynthesis Pathway	DHFR and PTR1	2-(4-((2,4-dichlorobenzyl)oxy)phenyl)-1H-benzo[d]imidazole	dual inhibitors of DHFR-TS and PTR1	[86]
*L. donovani*	Folate Biosynthesis Pathway	DHFR and PTR1	2-(4-((2,4-dichlorobenzyl)oxy)phenyl)-1H-benzo[d]imidazole-1H-benzo[d]oxazole	dual inhibitors of DHFR-TS and PTR1	[86]
*L. major*	Folate Biosynthesis Pathway	PTR1	Sophoraflavanone G	Inhibits PTR1 activity	[87]
*L. chagasi*	Folate Biosynthesis Pathway	DHFR and PTR1	2,4-diaminoquinazoline	dual inhibitors of DHFR-TS and PTR1	[88]
*L. braziliensis*, *L. Mexicana*, and *L. amazonensis*	Folate Biosynthesis Pathway	PTR1	2-arylquinazolin-4(3H) ones	Inhibits PTR1 activity	[92]
*L. infantum*	Trypanothione pathway	TR	[RDS 777] (6-(sec-butoxy)-2-((3-chlorophenyl) thio) pyrimidin-4-amine)	forms hydrogen bonds with the catalytic residues Glu466’, Cys57, and Cys52, limiting trypanothione binding and preventing its reduction	[99]
*L. donovani*	Trypanothione pathway	TR	trichloro [1,2-ethanediolato-O,O’]-tellurate (AS101)	Inhibits TR by forming thiol bonds with cysteine residues of TR., thus inducing ROS mediated apoptosis	[100]
*L. infantum*	Trypanothione pathway	TR	pyrrolopyrimidine	Disrupting the homodimeric interface trypanothione disulfide reductase	[101]
*L. infantum*	Trypanothione pathway	TR	5-6-5 imidazole-phenyl-thiazole-helix-mimetic scaffolds	Disrupting the homodimeric interface trypanothione disulfide reductase	[101]
*L. infantum*	Trypanothione pathway	TR	Triazole-phenyl-thiazoles	Disrupting the homodimeric interface trypanothione disulfide reductase	[102]
*L. braziliensis*	Trypanothione pathway	TR	resveratrol analogues	Induce ROS by inhibiting TR activity	[103]
*L. mexicana*	Trypanothione pathway	TR	N-(6-quinolinemethyl)-3-pyrazole carboxamide	formation of hydrogen bonds with the active site of TR	[105]
*L. donovani*	Trypanothione pathway	TR	β-sitosterolCCL	Inhibit TR activity	[108]
*L. infantum*	Trypanothione pathway	TR	(-)-Epigallocatechin 3-O-gallate (EGCG)	a competitive inhibitor of the trypanothione substrate.	[110]
*L. infantum*	Trypanothione pathway	TR	3-Methoxycarpachromene	Inhibits TR activity	[107]
*L. donovani*	Hypusine pathway	spermidine synthase	hypericin	decrease spermidine availability and induce ROS	[121]

## Data Availability

Not applicable.
